# Cooperative Monostatic and Bistatic Measurements for Low-Altitude UAV ISAC: System Implementation and Channel Characterization

**DOI:** 10.3390/s26165015

**Published:** 2026-08-07

**Authors:** Nan Ming, Hanwen Xu, Kai Mao, Hanpeng Li, Mingqi Guo, Xiaomin Chen, Qiuming Zhu

**Affiliations:** 1 The Key Laboratory of Dynamic Cognitive System of Electromagnetic Spectrum Space, College of Electronic and Information Engineering, Nanjing University of Aeronautics and Astronautics, Nanjing 211106, China; mingnan@nuaa.edu.cn (N.M.); xuhanwen@nuaa.edu.cn (H.X.); lihanpeng@nuaa.edu.cn (H.L.); mingqi_guo@nuaa.edu.cn (M.G.); zhuqiuming@nuaa.edu.cn (Q.Z.); 2 Centre for Wireless Innovation (CWI), Queen’s University Belfast, Belfast BT3 9DT, UK; k.mao@qub.ac.uk

**Keywords:** low-altitude UAV, ISAC, cooperative monostatic–bistatic measurement, channel characterization

## Abstract

Low-altitude unmanned aerial vehicle (UAV)-integrated sensing and communication (ISAC) channels are governed by rapidly evolving multipath. These dynamics arise from UAV motion, air–ground geometry, and scene-dependent scatterers, yet field evidence comparing monostatic and bistatic sensing links remains limited. We develop a cooperative monostatic–bistatic measurement system for low-altitude UAV ISAC channel sounding. The system integrates a UAV-borne sensing node, a ground node, synchronized acquisition, and an offline processing chain. It enables UAV-borne monostatic sensing and air–ground bistatic sensing to be measured within the same low-altitude urban scenario. A field measurement campaign is conducted along a representative route containing buildings, trees, roadside facilities, and open ground. From the measured in-phase/quadrature (IQ) data, the system extracts channel impulse responses (CIRs) and power delay profiles (PDPs) as primary measurement products. Delay–Doppler processing, multipath-component extraction, and trajectory analysis are applied to compare link-dependent propagation behavior under matched environmental conditions. The measurement results demonstrate that the proposed platform can jointly capture monostatic and bistatic ISAC channel responses and support controlled and synchronized low-altitude UAV channel measurement experiments. This system provides an experimental basis for UAV ISAC channel modeling, measurement-platform assessment, and subsequent sensing algorithm evaluation.

## 1. Introduction

Low-altitude unmanned aerial vehicles (UAVs) are becoming important airborne nodes in future wireless networks, where they are expected to support flexible communication coverage and environment-aware services [1]. Integrated sensing and communication (ISAC) provides a promising framework for this objective by allowing communication and sensing functions to share spectrum, hardware, and waveform resources [2]. Joint communication and radio sensing has also been regarded as a key enabler for perceptive mobile networks [3]. Recent studies have further shown that large AI models and large language models are becoming important enablers for intelligent low-altitude UAV systems, supporting environment understanding, task reasoning, mission planning, swarm coordination, and adaptive UAV communication and operation [4,5]. These emerging intelligent UAV applications increase the need for reliable low-altitude channel measurements, because data-driven and model-assisted UAV ISAC systems require realistic propagation evidence for system design, validation, and deployment. However, low-altitude UAV ISAC links are not simple extensions of terrestrial links, because their propagation is jointly shaped by three-dimensional mobility, flight altitude, trajectory, buildings, trees, road facilities, and ground reflections. Measurement studies have shown that these factors can cause strong variations in channel impulse response, delay dispersion, and Doppler behavior in air-to-ground UAV channels [6,7]. When monostatic and bistatic sensing links coexist, the same environment is observed from different geometric perspectives. Therefore, coordinated monostatic–bistatic channel measurement is needed to obtain comparable observations under matched environmental conditions. Such measurements are important for low-altitude UAV ISAC channel modeling, waveform design, and sensing-performance evaluation.

Existing studies provide important foundations for ISAC channel measurement and UAV air-to-ground channel characterization. Liu et al. discussed ISAC as a dual-functional wireless-network framework and emphasized that sensing and communication performance are jointly affected by propagation channels, waveform design, and hardware sharing [2]. Zhang et al. further surveyed joint communication and radar sensing in mobile networks, showing that realistic propagation characterization is essential for evaluating sensing-assisted and communication-assisted network functions [3]. For UAV scenarios, Khawaja et al. and Mao et al. reviewed air-to-ground propagation models, channel-sounding technologies, and UAV-assisted measurement campaigns [1,8]. Mao et al. further developed a UAV-aided real-time channel sounder for highly dynamic nonstationary air-to-ground scenarios [9]. Rodriguez-Pineiro et al. measured low-height UAV channels in realistic cellular deployments and analyzed path loss, shadow fading, delay spread, Doppler spread, and K-factor statistics [10]. Cai et al. extracted channel impulse responses from commercial LTE signals and characterized low-altitude air-to-ground multipath behavior [6], while Lyu et al. measured and modeled fixed-wing UAV air-to-ground channels at 2.7 GHz in a rural environment [11]. Mao et al. also proposed a measurement-driven cluster-power generation method for hybrid air-to-ground channel modeling [12]. Measurements at UWB and millimeter-wave bands have also been reported for UAV air-to-ground links [13,14,15]. These studies have established a solid basis for UAV communication-channel modeling and ISAC-oriented system design. However, most existing UAV channel measurements still focus on communication links and aggregate channel statistics. They do not directly capture how monostatic sensing and bistatic sensing observe the same low-altitude environment from different geometries. To the best of our knowledge, coordinated low-altitude UAV ISAC channel measurement with UAV-borne monostatic sensing and air–ground bistatic sensing under the same propagation environment has not yet been sufficiently investigated. Monostatic and bistatic sensing links differ in propagation geometry and interference mechanisms. In a monostatic configuration, the transmitter and receiver are located on the same platform, so the link is sensitive to local Doppler-active multipath near the UAV. The same configuration is also susceptible to transmit–receive coupling, platform reflections, and residual dominant components near zero Doppler. In a bistatic configuration, the transmitter and receiver are spatially separated, which reduces part of the monostatic self-interference and observes targets and environmental scatterers under a different geometry. Prior ISAC studies have shown that monostatic and bistatic sensing are complementary in observation geometry, sensing coverage, and interference behavior [16,17,18]. Cluster-based representations have also been widely used in millimeter-wave and MIMO channel modeling to describe multipath structures with similar delay, angular, and power-domain characteristics [19,20]. Related cluster-based methods have been applied to vehicular channel characterization [21,22]. However, Doppler-active multipath structures in synchronized monostatic and bistatic low-altitude ISAC measurements remain limited.

To address this gap, we develop a cooperative monostatic–bistatic low-altitude UAV ISAC channel measurement system and organize measurement activities in a representative urban scenario. The system integrates an aerial sensing node and a ground node, and supports both UAV-borne monostatic self-transmitting/self-receiving sensing and air–ground bistatic sensing under the same propagation environment. Based on the collected IQ data, a unified processing workflow is established to obtain channel impulse responses (CIRs), power delay profiles (PDPs), multipath components (MPCs), and Doppler information for both sensing links.

The main contributions of this paper are summarized as follows:

- A cooperative monostatic–bistatic measurement system is developed for low-altitude UAV ISAC channels. UAV-borne monostatic sensing and air–ground bistatic sensing are measured in the same road environment, providing matched observations for different sensing geometries.
- A measurement and processing workflow is designed to extract CIRs, PDPs, MPCs, and Doppler information from the collected IQ data. For the monostatic link, residual components associated with the dominant UAV self-transmitting/self-receiving response cluster are further suppressed to improve the observation of effective propagation components.
- Field measurements are conducted in a representative low-altitude road scenario, and the two sensing links are compared using delay–Doppler scattering distribution, equivalent range/path-length Doppler occupancy, and multipath-track persistence. The results provide experimental evidence for link-dependent propagation behavior in low-altitude UAV ISAC channels.

The remainder of this paper is organized as follows. Section 2 describes the cooperative monostatic–bistatic measurement system and field-measurement workflow. Section 3 presents the data-processing and channel-characterization methods. Section 4 reports the measurement campaign and discusses the measured channel characteristics. Section 5 concludes the paper.

## 2. Developed Channel Measurement System

The developed measurement platform is designed to acquire synchronized monostatic and bistatic UAV ISAC channel responses under the same propagation environment. Its hardware configuration, timing synchronization, and acquisition workflow are introduced below.

### 2.1. System Setup

**Figure 1:**
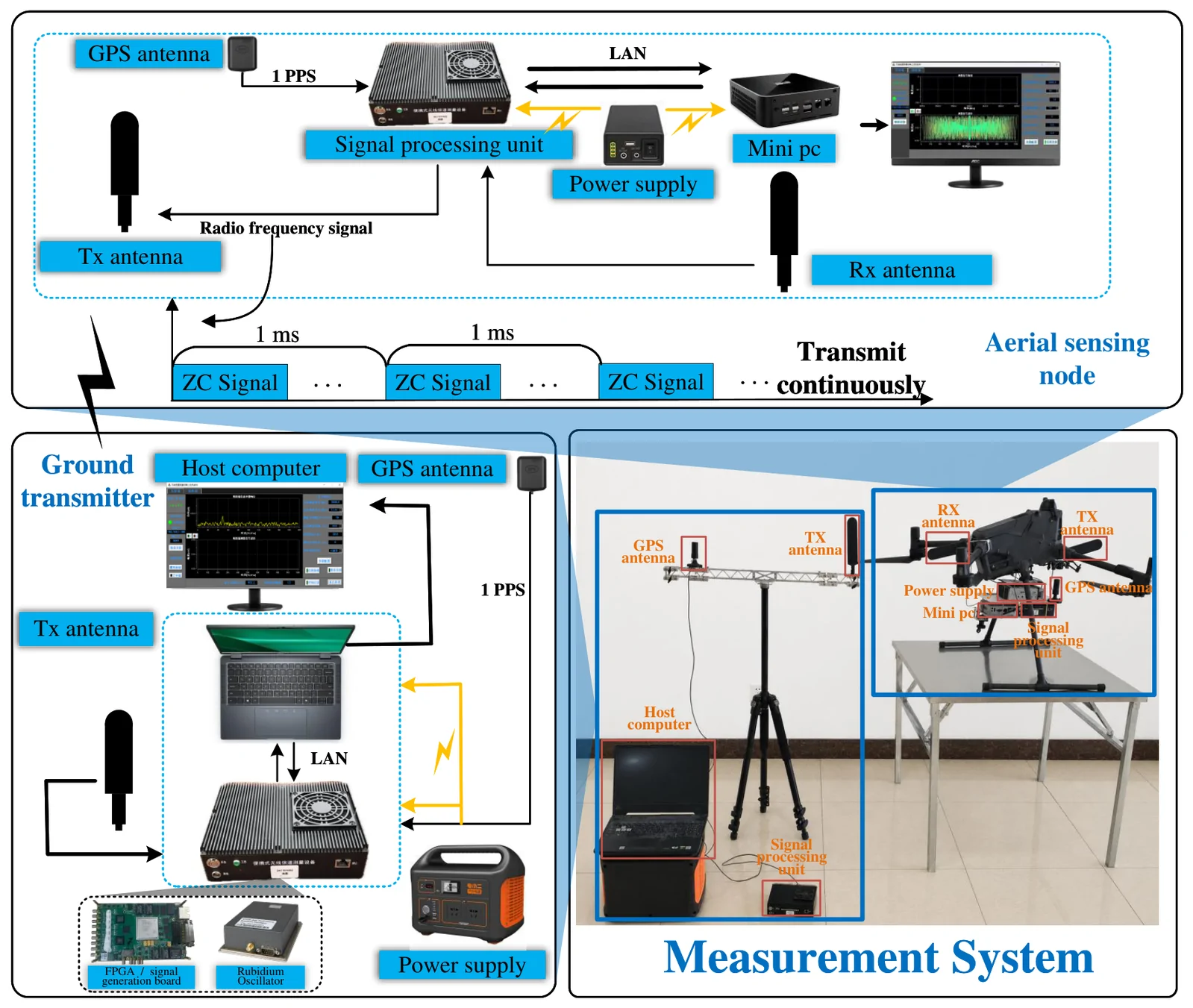
Prototype of the developed ISAC channel measurement system.

Figure 1 shows the developed cooperative monostatic–bistatic ISAC channel measurement system. The system consists of an aerial sensing node mounted on the UAV platform and a fixed ground transmitting node. The aerial node supports monostatic self-transmitting/self-receiving sensing and also receives the bistatic sounding signal transmitted by the ground node. The ground node provides the air–ground bistatic sounding signal, while the aerial node records the received IQ data for subsequent channel processing. Both nodes are synchronized by Global Positioning System (GPS) pulse-per-second (PPS) signals and GPS time information, which provide a common time reference for the monostatic and bistatic measurements. A rubidium clock is used to generate a high-stability local reference clock, improving timing stability during channel sounding. The synchronized configuration allows the two sensing links to be measured under a consistent time reference.

The aerial sensing node includes a GPS antenna/module, a signal-processing unit, radio frequency (RF) transmitting and receiving chains, transmit and receive antennas, an onboard mini PC, and a portable power supply. The signal-processing unit generates the monostatic sounding signal and processes the received baseband IQ data. The onboard mini PC configures the measurement parameters, buffers IQ frames, and stores the data locally.

The ground transmitting node includes a GPS antenna/module, a signal-generation unit, an RF transmitting chain, a transmit antenna, a host computer, and a portable power supply. It generates and transmits the bistatic sounding signal toward the aerial node. Together, the two synchronized nodes form a cooperative measurement platform for low-altitude UAV monostatic and bistatic ISAC channel sounding.

It should be clarified that the bistatic sensing link in this work is not treated as a pure one-way air-to-ground communication channel. The developed system simultaneously records two sensing-related channel responses at the same UAV-borne receiver: the UAV-borne monostatic response generated by the aerial transmitter and the ground-to-UAV bistatic response generated by the spatially separated ground transmitter. For the bistatic link, the received signal includes not only the direct ground-to-UAV propagation component, but also the Tx–scatterer–Rx components reflected or scattered by surrounding objects such as buildings, roads, vegetation, ground surfaces, and the nearby water area. Let $r_{G}$, $r_{U}(t)$, and $r_{S}$ denote the positions of the ground transmitter, UAV-borne receiver, and an environmental scatterer, respectively. The direct bistatic path length is $L_{\mathrm{dir}}\left(t\right)=∥r_{U}\left(t\right)-r_{G}∥$, whereas a reflected or scattered path has the bistatic length $L_{\mathrm{scat}}\left(t\right)=∥r_{S}-r_{G}∥\;+\;∥r_{U}\left(t\right)-r_{S}∥$. In this work, the bistatic link is therefore used for channel and multipath characterization under a separated-transmitter/same-receiver geometry, rather than for target-specific bistatic radar localization. Therefore, the bistatic measurement provides a separated-transmitter sensing geometry that is complementary to the UAV-borne monostatic link under the same receiving platform and measurement time reference.

To ensure comparable monostatic and bistatic measurements, the two nodes were operated with the same frequency band, bandwidth, sounding waveform, and antenna configuration. GPS PPS and GPS time information provided the common timing basis for synchronized sounding and frame-level data alignment. The main system parameters used in the measurement campaign are summarized in Table 1.

### 2.2. System Workflow

**Figure 2:**
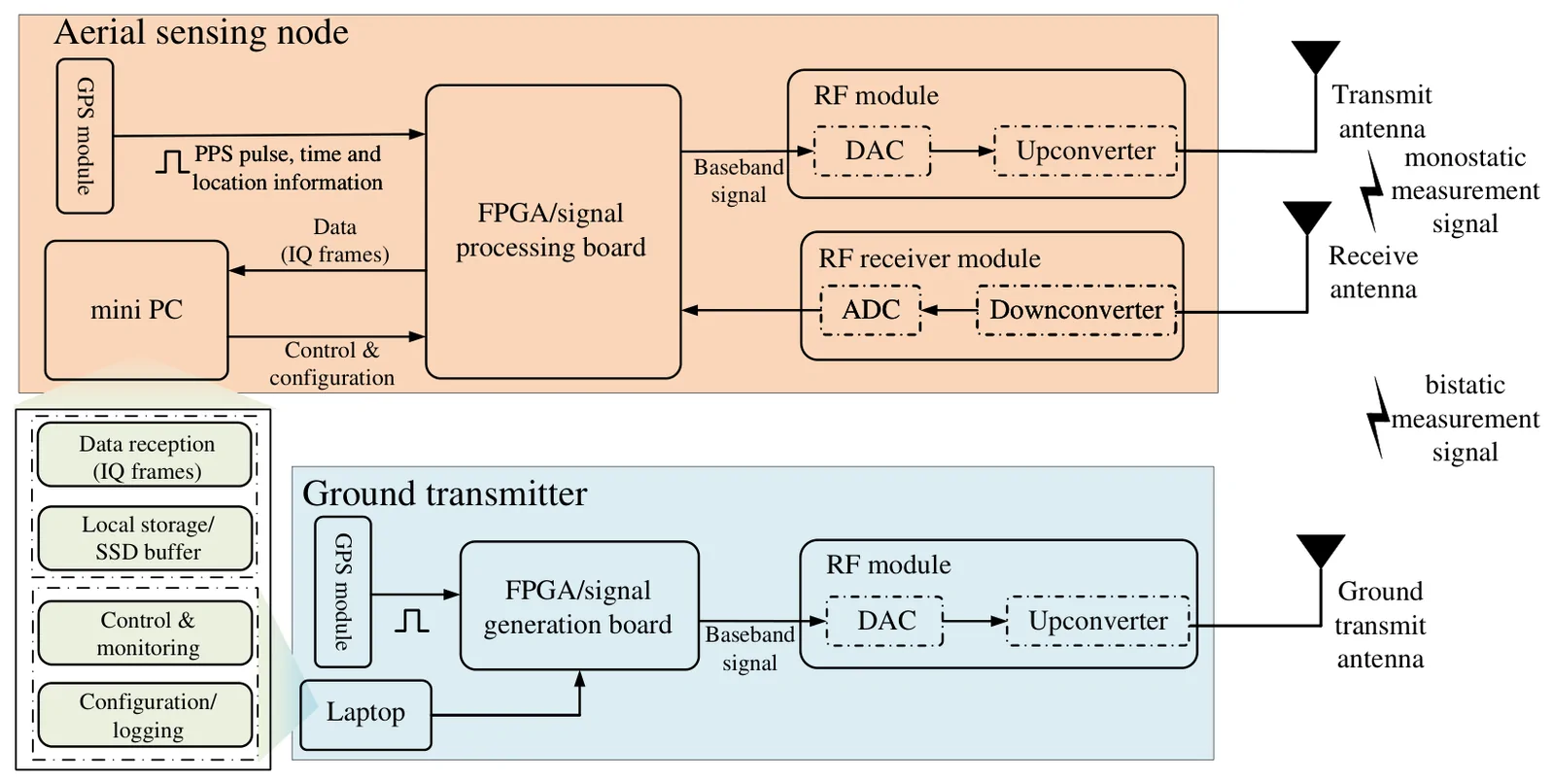
Workflow of the developed ISAC channel measurement system.

Figure 2 illustrates the signal flow and acquisition workflow of the developed measurement system. Under GPS PPS synchronization, the aerial sensing node generates the monostatic sounding signal, while the ground transmitting node generates the bistatic sounding signal. In the aerial node, the FPGA/signal-processing board outputs the baseband signal, which is converted by the Digital-to-Analog Converter (DAC) and up-converter before being radiated by the onboard transmit antenna. The onboard receive antenna captures the monostatic echo as well as the signal transmitted from the ground node. The received RF signal is first down-converted and then digitized by the Analog-to-Digital Converter (ADC) to obtain baseband IQ data.

In the ground transmitting node, the GPS module provides PPS timing for the FPGA/signal-generation board. The generated baseband bistatic sounding signal is converted to RF through the DAC and up-converter and then radiated by the ground transmit antenna toward the UAV-borne receiver. The host laptop configures the ground transmitter and records the operating status.

The received IQ frames at the aerial node are transferred to the onboard mini PC for data reception, local storage, configuration, and status logging. These stored IQ data are then used for subsequent CIR recovery, PDP calculation, MPC extraction, Doppler estimation, and channel-feature analysis.

## 3. Data Processing and Channel Characterization

The recorded IQ data are processed through a unified workflow so that the monostatic and bistatic links can be compared under consistent criteria. The workflow includes sounding-sequence correlation, calibrated CIR/PDP extraction, MPC detection, Doppler estimation, trajectory tracking, and statistical characterization.

### 3.1. Sounding Sequence Design and Calibrated Channel Response Extraction

Two Zadoff–Chu (ZC) sequences are used to support concurrent monostatic and bistatic channel sounding. The first sequence is assigned to the aerial monostatic link, and the second sequence is assigned to the air–ground bistatic link. For a sequence length *N*, the ZC sequence with root index *u* is expressed as

(1) $$x_{u}\left[k\right]=\exp\left(-j\pi uk^{2}/N\right),\;k=0,1,\ldots ,N-1.$$

The two sequences are denoted as $x_{M}\left[k\right]=x_{u_{1}}\left[k\right]$ and $x_{B}\left[k\right]=x_{u_{2}}\left[k\right]$, where the subscripts M and B denote the monostatic and bistatic links, respectively. In the measurement campaign, the root indices were set to $u_{1}=1$ for the monostatic sequence and $u_{2}=29$ for the bistatic sequence. Since ZC sequences have impulse-like cyclic autocorrelation, they are suitable for delay-domain channel sounding. To reduce mutual interference between the two sounding signals, the root index of the bistatic sequence is selected by minimizing the maximum cyclic cross-correlation with the monostatic sequence:

(2) $$u_{2}=\arg\min_{u\in U,\;u\neq u_{1}}\max_{l}\left|\frac{1}{N}\sum_{k=0}^{N-1}x_{u_{1}}\left[k\right]x_{u}^{*}\left[\left(k-l\right)\;\mathrm{mod}\;N\right]\right|,$$

where U={u∈{1,…,N−1}:gcd(u,N)=1} denotes the candidate set of valid ZC root indices. This quasi-orthogonal design enables the receiver to separate the monostatic and bistatic responses by correlating the received signal with the corresponding local reference sequence.

For the *n*-th received frame, the baseband samples at the aerial receiver contain both the monostatic echo and the bistatic air–ground signal. The received signal is modeled as

(3) $$r\left[n,k\right]=\sum_{s\in \{M,B\}}\sum_{l}h_{s}^{\mathrm{raw}}\left[n,l\right]\;x_{s}\left[\left(k-l\right)\;\mathrm{mod}\;N\right]+w\left[n,k\right],$$

where $h_{s}^{\mathrm{raw}}\left[n,l\right]$ is the raw channel impulse response (CIR) of link *s*, and w[n,k] denotes noise and residual interference.

The raw CIRs of the two links are extracted by sliding correlation with the corresponding local reference sequence:

(4) $$h_{s}^{\mathrm{raw}}\left[n,l\right]=\frac{1}{N}\sum_{k=0}^{N-1}r\left[n,k\right]\;x_{s}^{*}\left[\left(k-l\right)\;\mathrm{mod}\;N\right],\;s\in \left\{M,B\right\}.$$

In the measurement campaign, the ADC sampling rate was 100 Msamples/s, the sounding bandwidth was 50 MHz, and the ZC sequence length was set to N=1024. The FFT size used for the frequency-domain implementation of the sliding correlation was 4096, and the extracted CIR contained 1024 delay bins. The delay resolution is therefore

(5) $$\Delta \tau =\frac{1}{B}=\frac{1}{50\times 10^{6}}=20\;\mathrm{ns}.$$

Accordingly, the corresponding monostatic range resolution is

(6) $$\Delta R_{M}=\frac{c\Delta \tau }{2}=\frac{c}{2B}\approx 2.998\;m,$$

which is approximately 3 m. For the bistatic link, the same delay resolution corresponds to a path-length resolution of cΔτ=c/B≈5.996m because the bistatic delay represents a one-way propagation path length.

The raw CIR includes both the wireless propagation response and the response of the measurement system, including cables, RF modules, converters, filters, and fixed system delay. To remove the system response, a back-to-back (B2B) calibration is performed before channel measurement. In the B2B calibration, the transmitter output is directly connected to the receiver input through a cable and attenuator, bypassing the wireless propagation channel. The measured B2B response therefore represents the equivalent system response of the measurement chain. Let $g_{B2B}\left[l\right]$ denote the measured B2B impulse response and $G_{B2B}\left[q\right]$ denote its frequency-domain representation. The calibrated CIR is obtained by frequency-domain de-embedding:

(7) $$\begin{array}{cc} H_{s}^{\mathrm{raw}}\left[n,q\right] & =F\{h_{s}^{\mathrm{raw}}\left[n,l\right]\},\\ H_{s}\left[n,q\right] & =\frac{H_{s}^{\mathrm{raw}}\left[n,q\right]G_{B2B}^{*}\left[q\right]}{|G_{B2B}{\left[q\right]|}^{2}+\epsilon },\\ h_{s}\left[n,l\right] & =F^{-1}\left\{H_{s}\left[n,q\right]\right\},\;s\in \left\{M,B\right\}, \end{array}$$

where F{·} and $F^{-1}\left\{\cdot \right\}$ denote the discrete Fourier transform and inverse discrete Fourier transform, respectively, and ϵ is a small regularization factor used to avoid numerical instability at frequency bins with weak B2B response.

The calibrated power delay profile (PDP) is calculated as

(8) $$P_{s}\left[n,l\right]={\left|h_{s}\left[n,l\right]\right|}^{2},\;s\in \left\{M,B\right\}.$$

The calibrated CIRs and PDPs provide the basis for subsequent MPC extraction and Doppler analysis.

### 3.2. Multipath-Component Extraction for Monostatic and Bistatic Links

MPC extraction is performed from the calibrated PDPs of the two links. For each link s∈{M,B}, adaptive peak detection is first applied to obtain candidate MPCs. The initial candidate MPC index set is defined as

(9) $$C_{s}^{\left(0\right)}=\left\{i=\left(n,l\right)|P_{s}\left[n,l\right]>\Gamma_{s}\left[n,l\right],\;l\in \mathrm{Peak}\left(P_{s}\left[n,:\right]\right)\right\},\;s\in \left\{M,B\right\},$$

where $\Gamma_{s}\left[n,l\right]$ is the local adaptive threshold, and $\tau_{l}$ is the delay of the *l*-th delay bin. For each candidate i=(n,l), the corresponding observation time, delay, and received power are assigned as $t_{s,i}=t_{n}$, $\tau_{s,i}=\tau_{l}$, and $P_{s,i}=P_{s}\left[n,l\right]$.

For each indexed candidate MPC *i*, the Doppler frequency ${\hat{\nu }}_{s,i}$ is estimated from the slow-time variation of the calibrated CIR $h_{s}\left[n,l_{i}\right]$ at the corresponding delay bin $l_{i}$. The subsequent screening differs between the two links because the dominant quasistatic component has different physical meanings in the monostatic and bistatic configurations. In the monostatic link, the co-located UAV-borne transmitter and receiver may produce direct leakage, antenna coupling, platform-related reflections, and residual self-transmitting/self-receiving components. These components may form dominant quasistatic responses and are therefore suppressed at the cluster level to avoid masking Doppler-active multipath components. For the monostatic link, following the LSF-based peak detection and modified DBSCAN clustering procedure in [22], SO-CFAR used 24 training cells and 6 guard cells with a false-alarm probability of $3\times 10^{-3}$. The modified DBSCAN algorithm was applied in the delay–Doppler plane using the normalized Euclidean distance $d_{ij}^{\mathrm{DD}}=\sqrt{{\left(\left(\tau_{i}-\tau_{j}\right)/\tau_{g}\right)}^{2}+{\left(\left(\nu_{i}-\nu_{j}\right)/\nu_{g}\right)}^{2}}$, where $\tau_{g}=20$ ns and $\nu_{g}=6$ Hz. Two detected peaks were regarded as neighbors when $d_{ij}^{\mathrm{DD}}\leq 1.0$, and the minimum cluster size was set to three MPCs. For monostatic quasistatic component suppression, the DBSCAN clusters were characterized by their integrated power and power-weighted delay centroid. The quasistatic dominant cluster was defined as the shortest-delay dominant cluster associated with the UAV self-transmitting/self-receiving main path, and this cluster was removed at the cluster level. The remaining clusters were retained as Doppler-active monostatic MPCs. The resulting retained monostatic MPC set is denoted as $C_{M}$. In the bistatic link, near-zero Doppler components may correspond to valid air–ground propagation paths. Therefore, all detected effective MPCs are retained, namely $C_{B}=C_{B}^{\left(0\right)}$.

Each retained MPC is represented as

(10) $$z_{s,i}={\left[t_{s,i},\tau_{s,i},{\hat{\nu }}_{s,i},P_{s,i}\right]}^{T},\;i\in C_{s},\;s\in \left\{M,B\right\},$$

where $t_{s,i}$, $\tau_{s,i}$, ${\hat{\nu }}_{s,i}$, and $P_{s,i}$ denote the observation time, delay, Doppler shift, and received power of the *i*-th retained MPC in link *s*, respectively.

### 3.3. Doppler Clustering and Multipath Trajectory Tracking

After MPC extraction, the retained MPCs are organized in the time–delay–Doppler domain. MPCs are first mapped into a normalized feature space using the hop-interval time scale $T_{g}=64$ frames (0.64 s), together with the delay and Doppler scales defined above. Points satisfying the time, delay, and Doppler gate constraints are regarded as neighboring MPCs. Connected neighboring points form one cluster, while small isolated clusters are discarded.

For trajectory tracking, the delay of each retained MPC is converted into equivalent distance:

(11) $$d_{s,i}=\frac{c\tau_{s,i}}{\kappa_{s}},\;\kappa_{M}=2,\;\kappa_{B}=1,$$

where *c* is the speed of light. Thus, $d_{M,i}$ represents the monostatic equivalent target range, whereas $d_{B,i}$ represents the bistatic propagation path length.

Trajectory association is performed between adjacent time windows using distance and Doppler continuity. For an active trajectory *k* in link *s* and a candidate MPC *i*, the association cost is

(12) $$J_{s,k,i}=\frac{|d_{s,i}-{\hat{d}}_{s,k}|}{D_{g}}+\frac{|{\hat{\nu }}_{s,i}-{\hat{\nu }}_{s,k}^{\mathrm{last}}|}{\nu_{g}},$$

where ${\hat{d}}_{s,k}$ is the predicted distance of trajectory *k*, ${\hat{\nu }}_{s,k}^{\mathrm{last}}$ is the latest Doppler estimate of the trajectory, and $D_{g}$ is the distance association gate. The candidate with the minimum valid cost is associated with the trajectory. Unmatched MPCs initialize new trajectories, and trajectories without valid associations for more than two consecutive windows are terminated. The tracked trajectory is expressed as

(13) $$T_{s,k}={\left\{(t_{s,k,r},d_{s,k,r},{\hat{\nu }}_{s,k,r},P_{s,k,r})\right\}}_{r=1}^{N_{s,k}^{\mathrm{point}}},\;s\in \left\{M,B\right\}.$$

To improve reproducibility, the main processing parameters used for MPC extraction, Doppler estimation, time–delay–Doppler clustering, and trajectory tracking are summarized in Table 2.

### 3.4. Comparative Analysis Framework for Monostatic and Bistatic Channels

The monostatic and bistatic links are compared using the retained MPCs and tracked trajectories. The comparison focuses on how the two geometries emphasize different multipath features. The monostatic link highlights Doppler-active multipath after suppressing the dominant UAV self-transmitting/self-receiving response cluster, whereas the bistatic link retains stable air–ground components and longer path-length responses.

For RMS delay and Doppler spread comparison, the retained MPCs are grouped into time windows. Let $C_{s,m}$ denote the retained MPC set of link *s* in the *m*-th time window. MPC powers are converted from dB to the linear domain before statistical calculation. The RMS spread of variable $\alpha_{s,i}$ is calculated as

(14) $$\sigma_{\alpha ,s}\left[m\right]=\sqrt{\frac{\sum_{i\in C_{s,m}}P_{s,i}{\left(\alpha_{s,i}-{\bar{\alpha }}_{s}\left[m\right]\right)}^{2}}{\sum_{i\in C_{s,m}}P_{s,i}}},\;{\bar{\alpha }}_{s}\left[m\right]=\frac{\sum_{i\in C_{s,m}}P_{s,i}\alpha_{s,i}}{\sum_{i\in C_{s,m}}P_{s,i}}.$$

When $\alpha_{s,i}=\tau_{s,i}$, $\sigma_{\alpha ,s}\left[m\right]$ gives the RMS delay spread. When $\alpha_{s,i}={\hat{\nu }}_{s,i}$, it gives the RMS Doppler spread.

The range/path-length marginal occupancy is used to compare where MPCs appear along the equivalent range or path-length axis. Let $I_{s,m}\left(a\right)$ denote whether at least one MPC of link *s* appears in distance bin *a* during the *m*-th time window. The marginal occupancy is calculated as

(15) $$O_{s}\left(a\right)=\frac{1}{N_{\mathrm{win}}}\sum_{m=1}^{N_{\mathrm{win}}}I_{s,m}\left(a\right),$$

where $N_{\mathrm{win}}$ is the number of time windows. A larger value indicates that MPCs appear more frequently in the corresponding range or path-length region.

Finally, the track-duration distribution is used to compare the temporal stability of traceable multipath trajectories:

(16) $$T_{s,k}^{\mathrm{dur}}=T_{s,k}^{\mathrm{end}}-T_{s,k}^{\mathrm{start}},$$

where $T_{s,k}^{\mathrm{start}}$ and $T_{s,k}^{\mathrm{end}}$ are the start and end times of trajectory $T_{s,k}$. The median and interquartile range of $T_{s,k}^{\mathrm{dur}}$ are used to summarize the typical lifetime and dispersion of tracked trajectories. Together, RMS delay spread, RMS Doppler spread, marginal occupancy, and track-duration distribution provide the basis for the comparative analysis of the monostatic and bistatic channels.

## 4. Measurement Results and Analysis

Based on the processing framework described above, the measured channel responses are analyzed to reveal link-dependent propagation behavior. The measurement scenario is first introduced, followed by comparisons of CIR/PDP evolution, delay–Doppler characteristics, range/path-length occupancy, and multipath trajectory persistence.

### 4.1. Measurement Campaign

**Figure 3:**
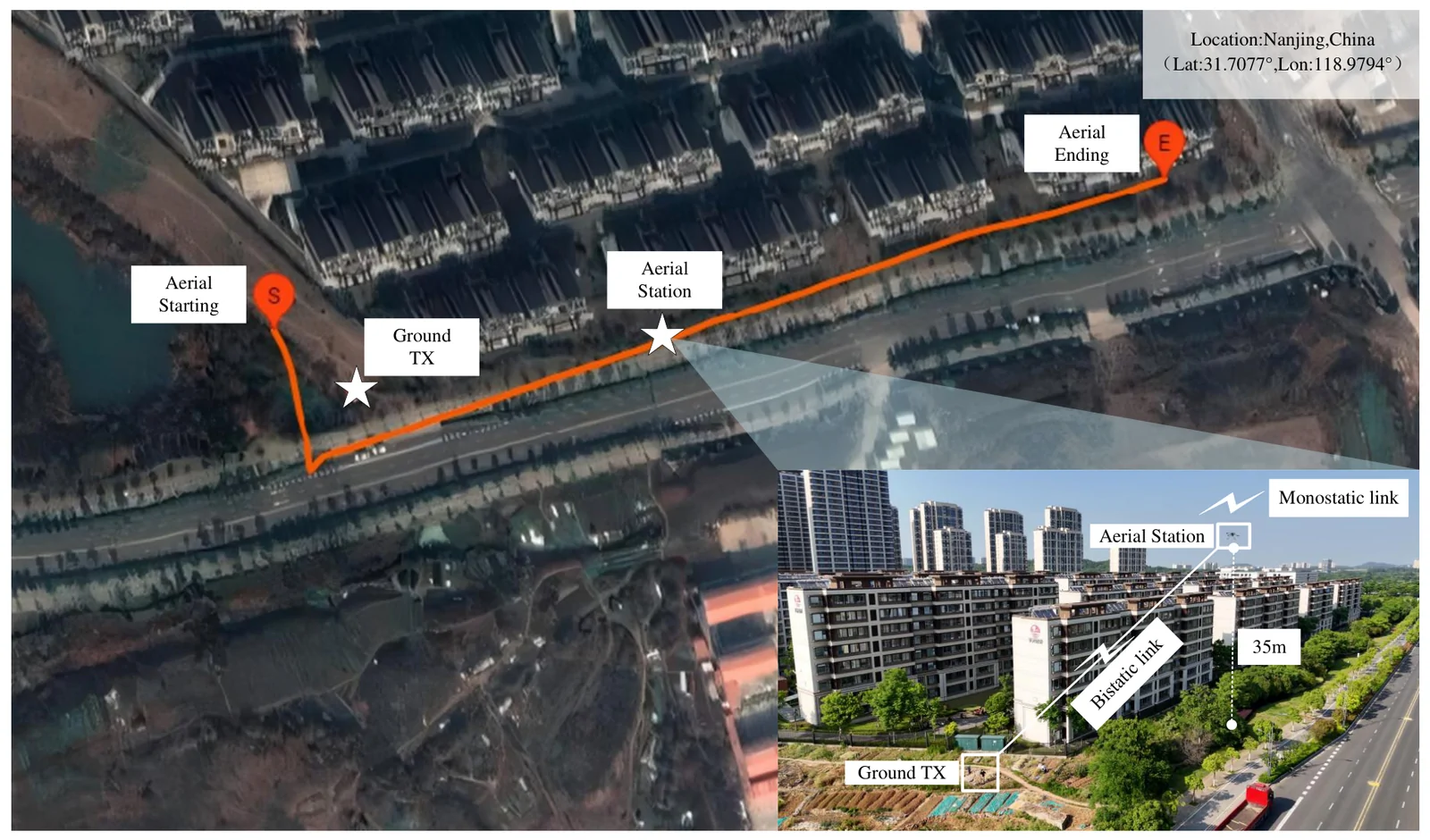
Measurement scenario.

The measurement campaign was conducted in a low-altitude road scenario. The fixed ground terminal was located at 31.7077 degrees latitude and 118.9794 degrees longitude, as shown in Figure 3. Buildings, trees, road facilities, green belts, and open ground surrounded the measurement area. These scatterers created reflection, scattering, and blockage conditions suitable for analyzing time-varying multipath in low-altitude UAV mobility.

During the measurement, the UAV served as the aerial mobile terminal and flew along a preset trajectory at an altitude of approximately 35 m. The ground terminal remained fixed as the reference node. The system operated at 3.2 GHz with a 10 ms frame interval, 50 MHz bandwidth, and a sounding-sequence length of 1024. Both devices were synchronized by GPS PPS signals to ensure inter-node time alignment and measurement accuracy. For validation of the flight condition, the UAV trajectory recorded by the GPS module was further checked against the DJI flight logs to verify the consistency of the flight route, altitude variation, and measurement timing. These records were used to support the interpretation of the measured delay/path-length evolution. However, no calibrated moving target or external LiDAR/camera-based ground-truth system was deployed in this campaign. Therefore, the reported results should be interpreted as channel-characterization observations and measurement-platform assessment rather than absolute target-localization validation.

### 4.2. Measurement Result Analysis

The measured results are interpreted from the perspective of link geometry, UAV motion, and environment-dependent multipath. For the monostatic link, polarization isolation and self-interference suppression determine whether weak local scattering components can be observed. For the bistatic link, the fixed ground terminal and the moving UAV form an air–ground propagation geometry in which stable direct and reflected paths can remain observable over longer path-length ranges. Therefore, the following analysis focuses on how these geometric differences lead to different PDP evolution, range/path-length occupancy, and trajectory-persistence characteristics.

**Figure 4:**
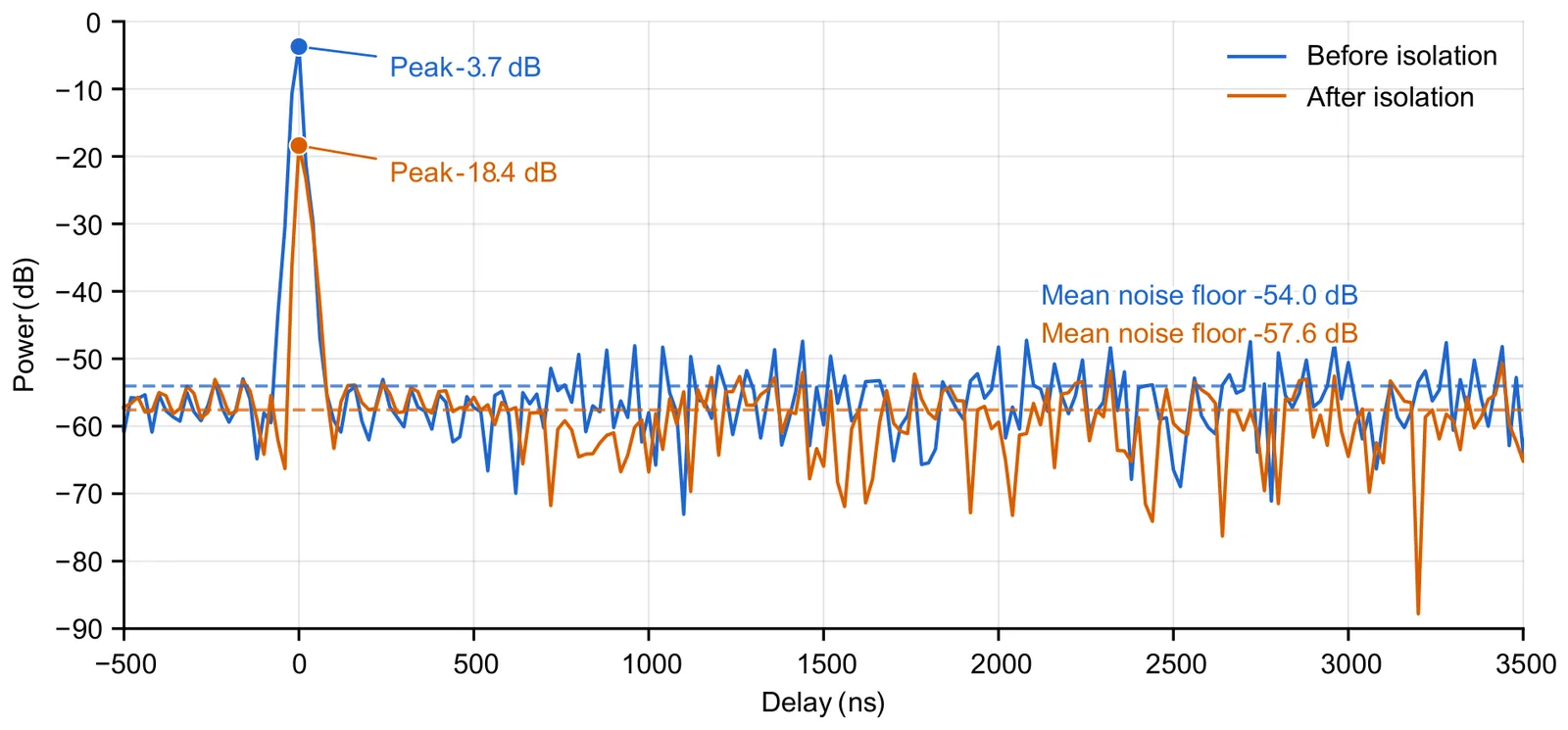
Single-frame CIR comparison of the monostatic UAV self-transmitting/self-receiving link before and after polarization–isolation optimization.

Figure 4 compares single-frame CIRs of the UAV self-transmitting/self-receiving link before and after polarization–isolation optimization. The two CIRs are aligned to the dominant-path peak for comparison. Before optimization, the CIR contains a strong near-zero-delay peak with a power of approximately −3.7 dB. After optimization, this peak decreases to −18.4 dB, indicating that direct transmitter-to-receiver leakage is substantially suppressed. The background-noise levels remain similar in the two cases, so the reduction in the dominant peak can be attributed mainly to the optimized antenna layout and chassis shielding. This suppression provides more favorable observation conditions for subsequent weak-scattering MPC detection.

**Figure 5:**
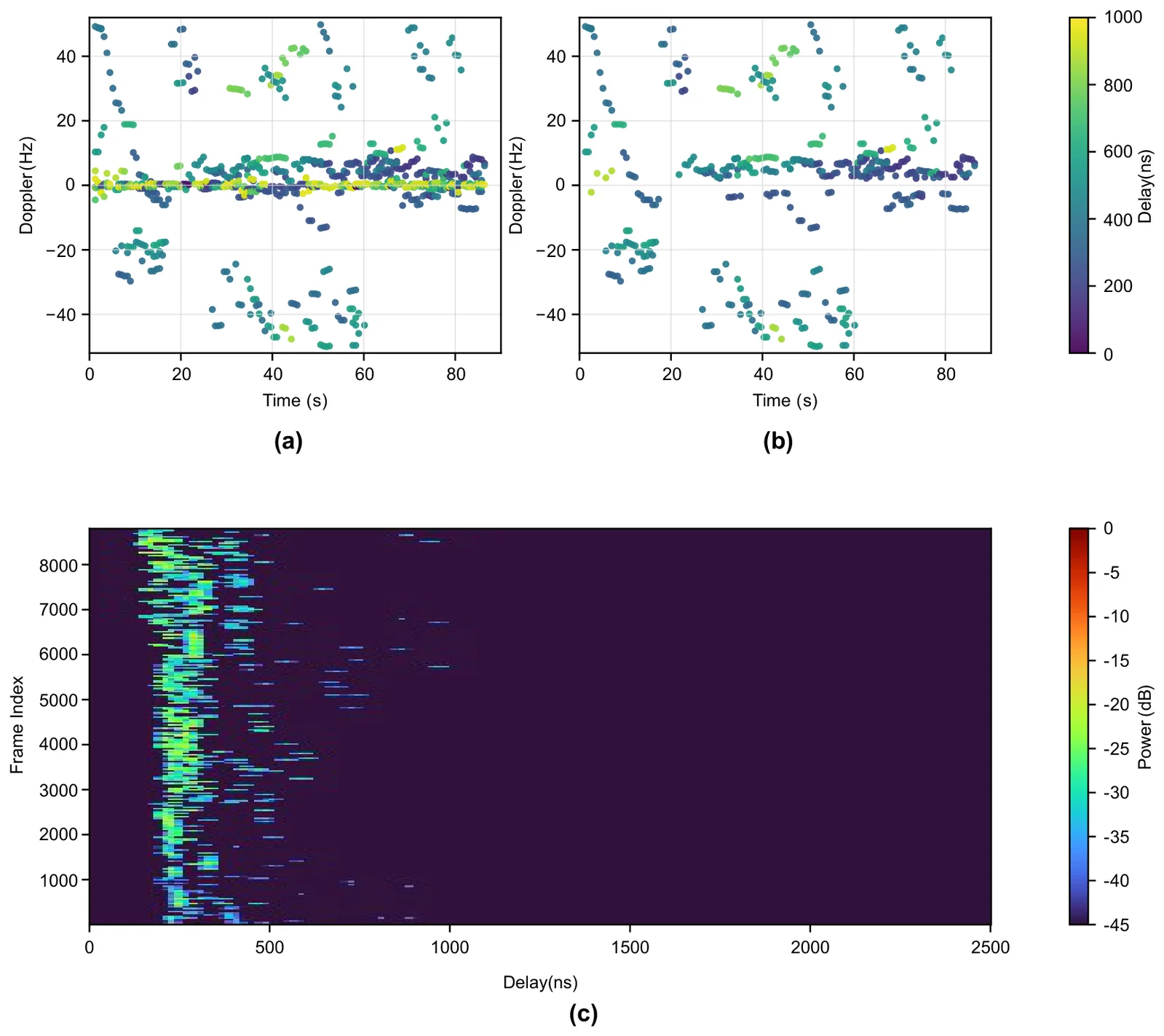
Monostatic MPC extraction and Doppler-active PDP reconstruction. (**a**) All detected monostatic MPCs. (**b**) Doppler-active monostatic MPCs after suppressing the UAV self-transmitting/self-receiving main path. (**c**) Monostatic Doppler-active, MPC-only PDP.

Figure 5 illustrates monostatic Doppler-active MPC extraction in the UAV self-transmitting/self-receiving link. Peak detection is first performed on the cleaned PDP to obtain candidate MPCs, and their Doppler frequencies are estimated, as shown in Figure 5a. The candidate set contains a dominant UAV self-transmitting/self-receiving main path, which is mainly associated with direct coupling, platform-related reflections, and residual leakage of the monostatic transceiver. This dominant main path is removed to suppress the self-transmitting/self-receiving response of the UAV-borne monostatic platform, as shown in Figure 5b. The retained components are referred to as Doppler-active MPCs relative to the moving UAV platform, rather than scatterers that are necessarily physically moving. The retained Doppler-active MPCs are finally mapped back to the time–delay plane to obtain the Doppler-active, MPC-only PDP in Figure 5c, which highlights the delay evolution of multipath components with observable Doppler shifts.

**Figure 6:**
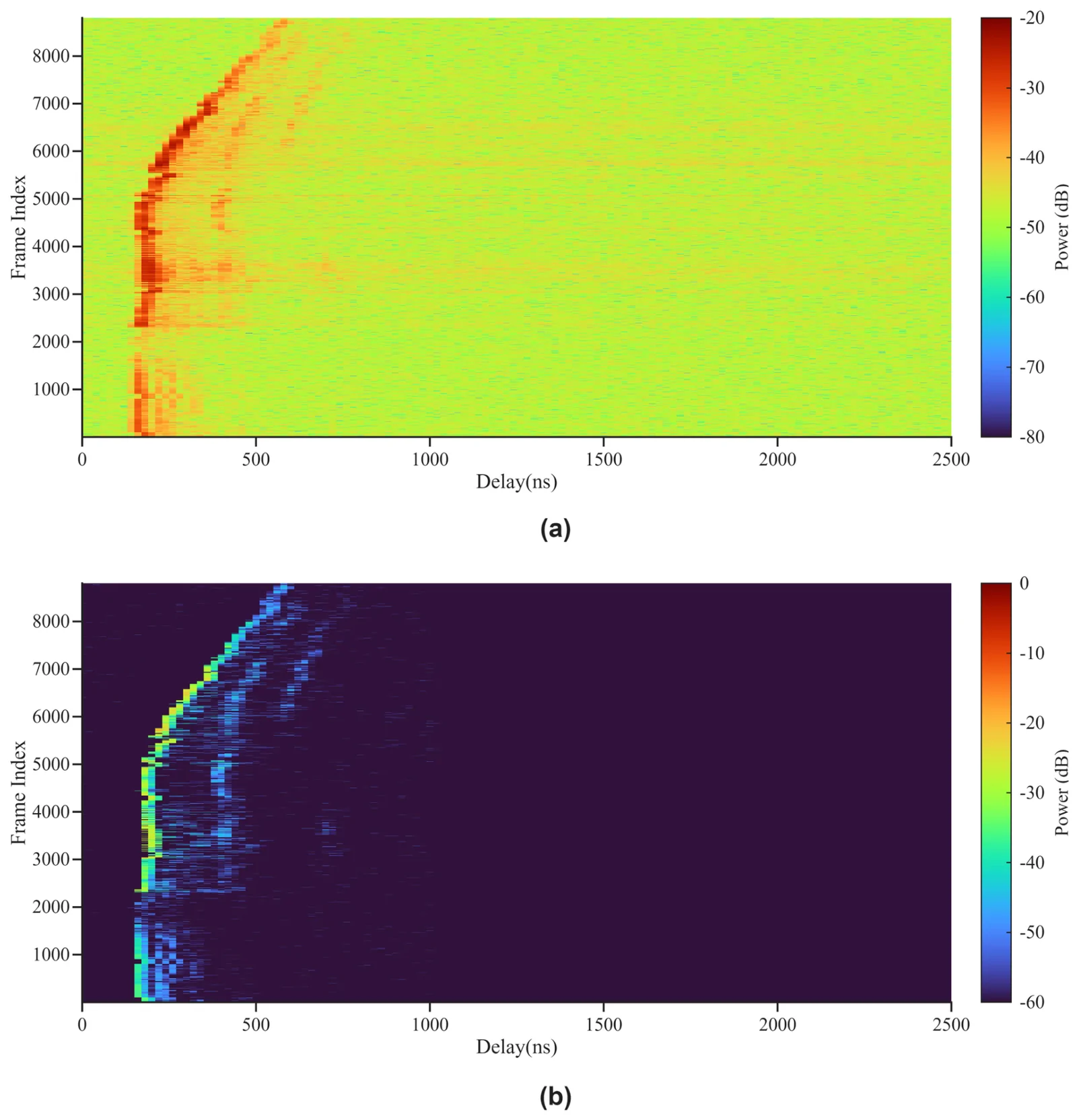
PDP processing result of the ground-to-UAV bistatic sensing link. (**a**) Calibrated bistatic PDP before final cleaning. (**b**) Cleaned absolute-delay bistatic PDP after fixed-delay correction and early-spurious-component suppression.

Figure 6 shows the PDP processing result of the bistatic sensing link. Figure 6a presents the PDP before the final cleaning step. A dominant propagation component is observed over most frames, corresponding to the stable ground-to-UAV propagation path. This component is retained because, in the bistatic link, it carries valid air–ground channel information rather than monostatic self-interference.

Figure 6b shows the cleaned PDP used for subsequent MPC extraction. After removing the fixed system delay and suppressing unreasonable early-arriving spurious components, the main propagation ridge remains visible. Background noise and isolated weak artifacts are largely reduced. The retained ridge varies gradually with the frame index, reflecting the delay variation introduced by UAV motion. Several weaker delayed components are also observed around the dominant ridge, indicating reflected or scattered paths in the ground-to-UAV air–ground channel.

This result shows that the bistatic PDP processing strategy differs from the monostatic case only in the post-detection cleaning stage. The two links use the same CFAR-based candidate-peak detection settings summarized in Table 2 for MPC extraction. In the monostatic link, the dominant quasistatic component is mainly associated with UAV self-transmitting/self-receiving leakage, antenna coupling, platform-related reflections, and residual leakage of the monostatic transceiver; therefore, it is suppressed at the cluster level to avoid masking Doppler-active multipath components. In the bistatic link, no comparable same-platform self-interference cluster exists; therefore, after system-delay correction, only early-arriving spurious artifacts caused by residual timing mismatch are suppressed, while valid ground-to-UAV propagation components are retained. Here, “spurious-component suppression” refers to the removal of such early-arriving artifacts rather than the removal of valid bistatic propagation paths.

**Figure 7:**
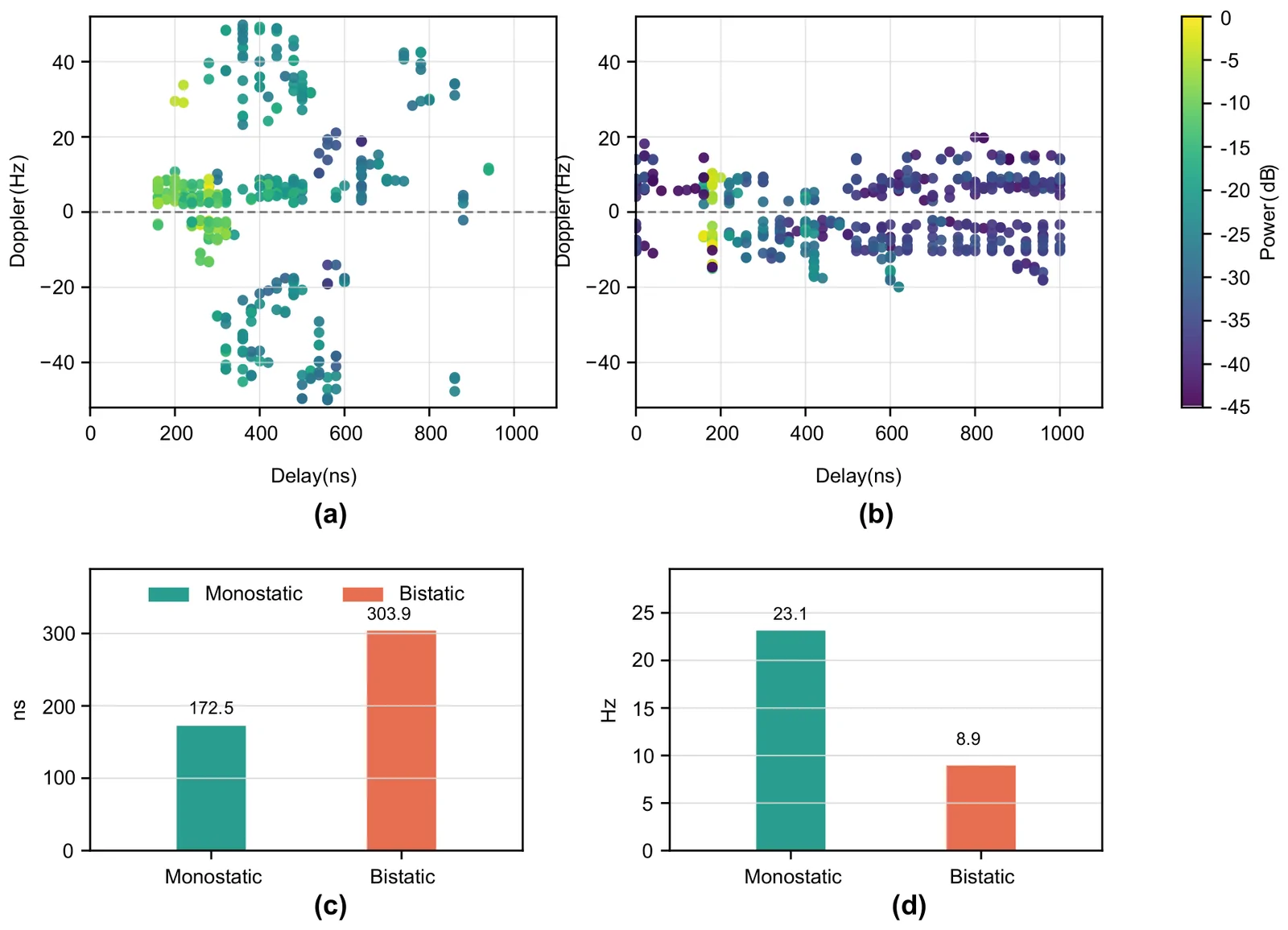
Comparison of delay–Doppler scattering and spread statistics for the two sensing links. (**a**) Delay–Doppler scattering distribution of the monostatic link. (**b**) Delay–Doppler scattering distribution of the bistatic link. (**c**) RMS delay spread comparison. (**d**) RMS Doppler spread comparison.

Figure 7 compares the delay–Doppler distributions of Doppler-active MPCs in the monostatic and bistatic links. Figure 7a,b show the detected MPC scatter distributions for the two links, respectively. The horizontal axis denotes calibrated delay, the vertical axis denotes Doppler frequency, and color represents MPC power. The same coordinate ranges and color scale are used in both plots to support direct comparison.

In Figure 7a, the monostatic MPCs are more dispersed along the Doppler dimension and include both positive and negative Doppler components with relatively large magnitudes. This distribution indicates that the monostatic link is sensitive to Doppler-active multipath around the moving UAV platform. The observed non-zero Doppler components may be caused by relative motion between the UAV-borne transceiver and surrounding objects, including both moving scatterers and static objects observed from the moving UAV platform. By contrast, the bistatic MPCs in Figure 7b are concentrated within a smaller Doppler range but span a broader delay range. The broader delay distribution is consistent with the air–ground geometry and longer propagation paths in the bistatic link.

Figure 7c,d quantify these differences. The RMS delay spread is approximately 172.5 ns for the monostatic link and 303.9 ns for the bistatic link. In contrast, the RMS Doppler spread is approximately 23.1 Hz for the monostatic link and 8.9 Hz for the bistatic link. Thus, the bistatic link shows a larger delay spread in this scenario, whereas the monostatic link shows a larger Doppler spread.

Compared with representative UAV air-to-ground measurement studies [6,10,11], the measured delay and Doppler spreads are of the same order of magnitude as previously reported low-altitude UAV results. However, because the compared studies differ in bandwidth, antenna configuration, trajectory, environment, transmitter power/amplification, and MPC extraction method, this comparison is used only as a qualitative physical reference. The main purpose of this work is the within-campaign comparison between the monostatic and bistatic links measured under the same system and processing framework.

**Figure 8:**
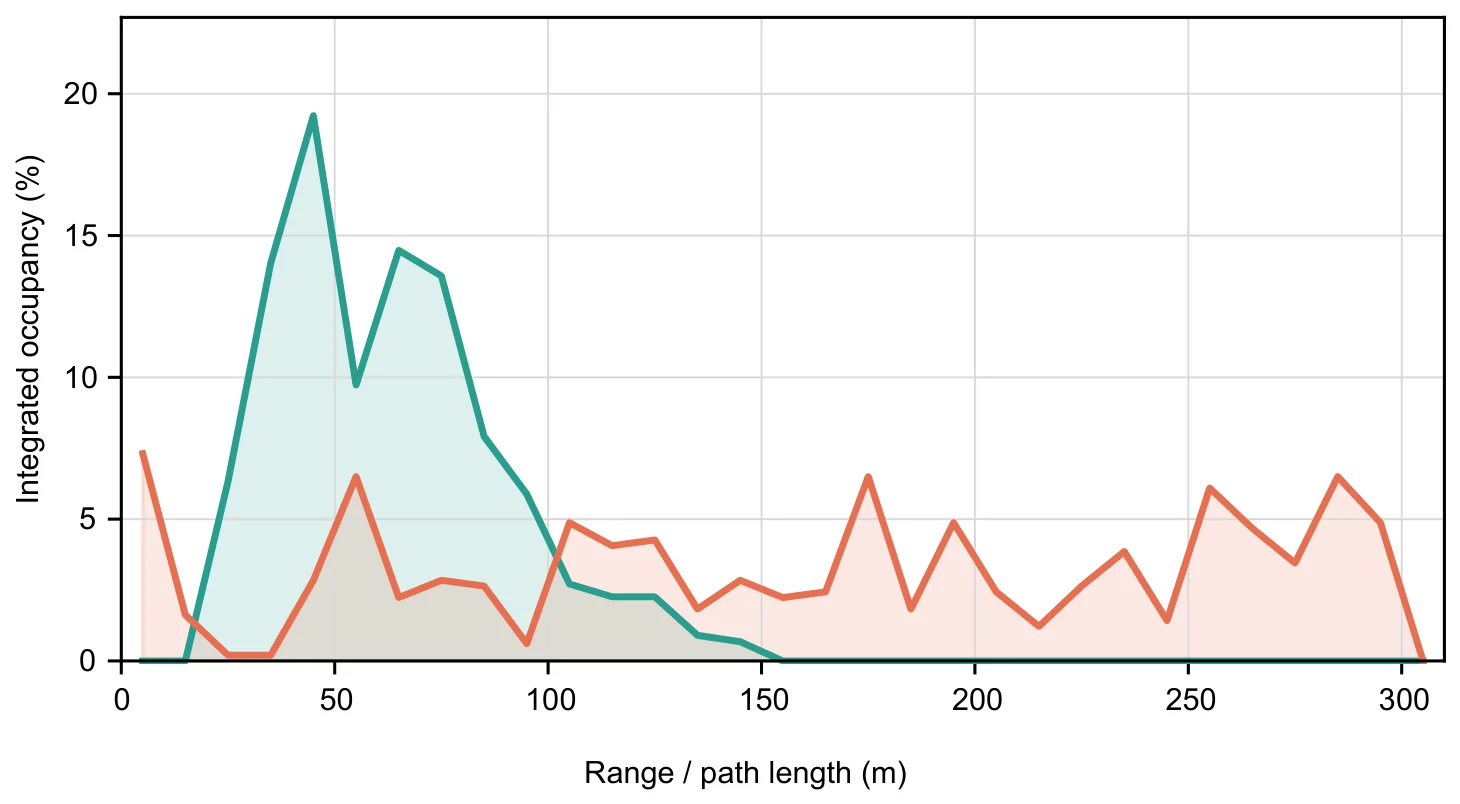
Range/path-length marginal occupancy comparison between the monostatic and bistatic links.

Figure 8 compares the occupancy distributions of Doppler-active MPCs along the equivalent target range or path-length coordinate. In the monostatic link, MPC occupancy is concentrated at shorter equivalent target ranges and peaks near 45 m, indicating that the response is mainly contributed by local scatterers near the UAV. In the bistatic link, the occupancy peak is lower but the distribution is broader, and Doppler-active MPCs are still detected at longer path lengths. This distribution shows that the bistatic link retains more detections over longer path lengths, whereas the monostatic link is dominated by shorter-range local scattering near the UAV platform. Physically, this difference results from the different observation geometries of the two links. The monostatic link mainly emphasizes scatterers located around the UAV platform because the transmitted and received signals share the same moving platform. In contrast, the bistatic link includes propagation between the fixed ground terminal and the moving UAV, so reflections and scattering along the air–ground path can remain observable over a wider path-length range.

**Figure 9:**
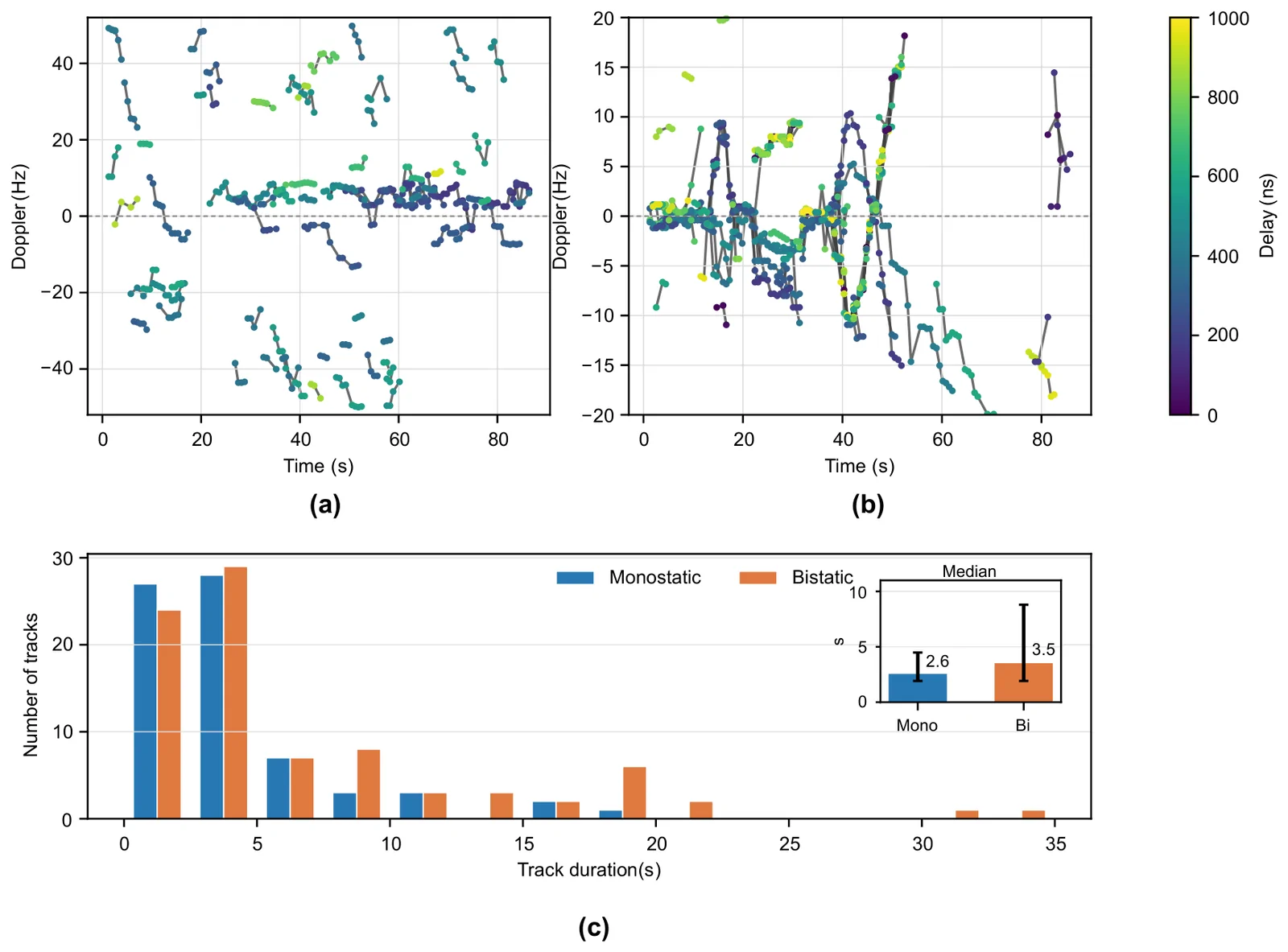
Multipath track persistence comparison between monostatic and bistatic links. (**a**) Monostatic time–Doppler trajectories colored by delay. (**b**) Bistatic time–Doppler trajectories colored by delay. (**c**) Track-duration distribution with median and interquartile range.

Figure 9 compares the temporal persistence of Doppler-active MPC trajectories in the two links. Figure 9a,b show the time–Doppler tracking results. Points and connecting lines represent the temporal evolution of the same multipath trajectory, and color indicates the corresponding MPC delay. Figure 9c summarizes the duration distribution of the trajectories and reports the median duration and interquartile range in the inset.

To provide a compact quantitative summary of the measured link-dependent differences, Table 3 reports descriptive aggregate statistics of the retained MPCs and trajectories.

The tracking results contain 71 effective multipath trajectories in the monostatic link. These trajectories occupy a wide Doppler range, and some have large positive or negative Doppler shifts, but most persist for short durations. The bistatic link contains 86 effective trajectories with a more concentrated Doppler distribution, and some trajectories show stronger temporal continuity. The median, mean, and maximum trajectory durations are 2.6 s, 4.0 s, and 19.8 s for the monostatic link, respectively. They increase to 3.5 s, 6.7 s, and 34.6 s for the bistatic link. In this measurement scenario, the monostatic link captures shorter-term multipath variations caused by local scatterers near the UAV, whereas the bistatic link provides more persistently observable scattering paths. This difference indicates that the monostatic link is more sensitive to short-term local variations around the UAV, whereas the bistatic link can preserve more stable air–ground propagation paths over time. The longer trajectory durations in the bistatic link are therefore consistent with the presence of persistent propagation paths between the fixed ground terminal and the moving UAV. The measured delay spread, Doppler spread, range/path-length occupancy, and trajectory persistence provide empirical statistics that can support 3GPP-style stochastic channel modeling by informing cluster delay, power, Doppler, and lifetime distributions. Full-model calibration across more environments and repeated flights is left for future work.

The observed propagation differences also provide practical implications for UAV ISAC waveform and system design. For the monostatic link, the broader Doppler distribution and stronger sensitivity to local UAV platform-related scattering suggest that waveform design should emphasize Doppler tolerance, self-interference suppression, and short-time observation capability. Shorter coherent processing intervals or more frequent Doppler updates may be preferred to track rapidly varying local components. In contrast, the bistatic link shows more concentrated Doppler behavior and longer trajectory persistence, indicating that waveform design should emphasize synchronization stability and sufficient observation duration for persistent air–ground paths. Therefore, monostatic and bistatic configurations should not necessarily use identical waveform and processing settings: the monostatic link is more suitable for local motion-sensitive sensing, whereas the bistatic link is more suitable for stable air–ground path monitoring and complementary environmental observation.

## 5. Conclusions

This study developed and tested a synchronized monostatic–bistatic channel measurement system for low-altitude UAV ISAC scenarios. By using GPS PPS synchronization, a common operating frequency, and a consistent processing workflow, the aerial monostatic and air–ground bistatic links were measured and compared under the same experimental framework. Rather than proposing a new ISAC or tracking algorithm, this work focuses on the implementation and measurement-level validation of a cooperative monostatic–bistatic measurement system and the experimental characterization of low-altitude UAV ISAC channels. The results show that the monostatic link is more sensitive to local Doppler-active multipath near the UAV after self-interference suppression, with a broader Doppler distribution, whereas the bistatic link captures a wider delay/path-length distribution and more persistent multipath trajectories between the fixed ground terminal and the moving UAV. The two links therefore emphasize different parts of the same environment: the monostatic link highlights local Doppler-active multipath observed from the moving UAV platform, while the bistatic link captures longer and more stable air–ground propagation paths. The conclusions are limited to the measured scenario, flight-height range, and trajectory, and future work should extend the measurements across more environments, altitudes, and repeated flights to support more general low-altitude ISAC channel models. 

## Figures and Tables

**Table 1:** Summary of the system parameters.

System Parameter	Value
Supported frequency band	450–6000 MHz
Bandwidth	50 MHz
Sounding sequence	Zadoff–Chu (ZC) sequence
Sequence length	Configurable (1024, 2048, 4096, etc.)
Sounding interval	10 ms
TX/RX antenna type	Omnidirectional
TX/RX antenna gain	2.5 dBi
TX/RX polarization configuration	Orthogonal linear polarization
TX/RX isolation arrangement	Shielded back-to-back TX/RX placement
Transmit power	32 dBm

**Table 2:** Main processing parameters for MPC extraction, Doppler estimation, time–delay–Doppler clustering, and trajectory tracking.

Processing Item	Parameter	Value/Setting
Local adaptive detection	CFAR method	SO-CFAR
Training-window setting	Training/guard cells	24/6
False-alarm control	False-alarm probability	$3\times 10^{-3}$
Candidate-peak search	Delay-bin search range	60–120 bins
Peak separation control	Minimum peak spacing	2 delay bins
Power-domain screening	Detection margin	Noise floor + 6 dB
Doppler estimation window	Window/hop length	256/64 frames
Doppler spectrum estimation	FFT size	512
Delay-tap integration	Tap half width	1 delay bin
Delay-scale normalization	Normalized delay scale	20 ns
Doppler-scale normalization	Normalized Doppler scale	6 Hz
Neighborhood definition	Normalized Euclidean radius	1.0
Cluster-size filtering	Minimum cluster size	3 MPCs
Trajectory validation	Minimum track length	3 detections
Monostatic trajectory association	Distance/Doppler gate	4.5 m/6 Hz
Bistatic trajectory association	Distance/Doppler gate	6 m/6 Hz
Time-scale normalization	Hop interval/time scale	64 frames (0.64 s)
Trajectory gap tolerance	Maximum missed consecutive windows	2 windows

**Table 3:** Descriptive aggregate statistics of retained MPCs and trajectories for the monostatic and bistatic links.

Statistic	Monostatic Link	Bistatic Link
Number of retained MPCs	442	492
Median delay (ns)	400.0	580.0
Delay IQR (ns)	280.0–500.0	300.0–840.0
RMS delay spread (ns)	172.5	303.9
Median |ν| (Hz)	8.4	7.8
|ν| IQR (Hz)	4.7–31.1	5.7–9.4
RMS Doppler spread (Hz)	23.1	8.9
Number of trajectories	71	86
Median trajectory duration (s)	2.6	3.5
Mean trajectory duration (s)	4.0	6.7
Maximum trajectory duration (s)	19.8	34.6

## Data Availability

The data generated and analyzed in this study are available from the corresponding author upon reasonable request.
